# Association of blood heavy metals with developmental delays and health status in children

**DOI:** 10.1038/srep43608

**Published:** 2017-03-02

**Authors:** Yu-Mei Hsueh, Chih-Ying Lee, Ssu-Ning Chien, Wei-Jen Chen, Horng-Sheng Shiue, Shiau-Rung Huang, Ming-I Lin, Shu-Chi Mu, Ru-Lan Hsieh

**Affiliations:** 1 Department of Family Medicine, Taipei Medical University Hospital, Taipei, Taiwan; 2 Department Public Health, School of Medicine, College of Medicine, Taipei Medical University, Taipei, Taiwan; 3 School of Medicine, College of Medicine, Taipei Medical University, Taipei, Taiwan; 4 School of Public Health, College of Public Health and Nutrition, Taipei Medical University, Taipei, Taiwan; 5 Department of Chinese Medicine, Chang Gung Memorial Hospital and Chang Gung University College of Medicine, Taoyuan, Taiwan; 6 Department of Pediatrics, Shin Kong Wu Ho-Su Memorial Hospital, Taipei, Taiwan; 7 Department of Physical Medicine and Rehabilitation, Shin Kong Wu Ho-Su Memorial Hospital, Taipei, Taiwan; 8 Department of Physical Medicine and Rehabilitation, School of Medicine, College of Medicine, Taipei Medical University, Taipei, Taiwan

## Abstract

The aim of this study was to evaluate the association of blood lead, mercury, and cadmium concentrations with developmental delays and to explore the association of these concentrations with the health status of children. This study recruited 89 children with developmental delays and 89 age- and sex-matched children with typical development. Their health status was evaluated using the Pediatric Quality of Life (PedsQL) Inventory for health-related quality of life (HRQOL) and the Pediatric Outcomes Data Collection Instrument for function. Family function was also evaluated. Blood lead, mercury, and cadmium concentrations were measured using inductively coupled mass spectrometry. The children with developmental delays had a considerably poorer HRQOL, lower functional performance and family function, and a higher blood lead concentration than those with typical development. The blood lead concentration had a significantly positive association with developmental delays [odds ratio (OR) = 1.54, p < 0.01] in a dose-response manner, and it negatively correlated with PedsQL scores (regression coefficient: −0. 47 to −0.53, p < 0.05) in all the children studied. The higher blood cadmium concentration showed a significantly positive association with developmental delays (OR = 2.24, for >1.0 μg/L vs. <0.6 μg/L, p < 0.05). The blood mercury concentration was not associated with developmental delays and health status.

Approximately 8–15% of children with physical, mental, communication, social, behavioural, or emotional developmental delays require special health assessment and care^1^. In Taiwan, children aged <7 years undergo regular physical check-ups and developmental screening a total of 7 times at paediatric clinics. This screening includes examination of gross and fine motor skills, communication, cognitive function, daily living performance, and social interaction. This regular assessment is nationally promoted by the Health Promotion Administration, Ministry of Health and Welfare. Children with suspected developmental delays are referred to early developmental intervention centres or clinics for further evaluation. Early developmental interventions, including evaluation and rehabilitation programmes, for children aged 0–6 years are covered by the Taiwan National Health Insurance programme, which was implemented in 1995. Under the Protection of Children and Youths Welfare and Rights Act (2012) and the Early Intervention Service Implementation Program for Children with Developmental Delays (2013), the government provides necessary counselling, referrals, placement, treatment, and other services to children aged 0–6 years with developmental delays and their families. Between 2000 and 2013, the prevalence of developmental delays in children aged 0–6 years increased from 3.12% to 9.31%^1^.

Exposure to heavy metals occurs through the ingestion, inhalation, and handling of contaminants in chemical products, industrial paints, building materials, fertilisers, nasal sprays, silver dental fillings, fish containing high mercury concentrations, and mercury-containing preservatives in vaccines^2^. Some heavy metals cause congenital and neurological defects, developmental delays, behavioural abnormalities, and learning disabilities and are considered potent developmental neurotoxins^2^,^3^,^4^. Among heavy metals, lead, mercury, and cadmium were ranked as the second, third, and seventh most hazardous substances on the priority list issued by the heavy metal subdivision of the Agency for Toxic Substances and Disease Registry in 2015^5^. Our previous study showed that the blood lead concentration and the arsenic methylation capacity independently contribute to the risk of developmental delays in preschool children in Taiwan^6^.

The health status of children has been increasingly acknowledged as a crucial outcome measure in health service evaluation and research^7^. According to the World Health Organization (WHO) International Classification of Functioning, Disability and Health, children’s health is dependent on dynamic interactions among functional, behavioural, environmental, and social factors and health-related quality of life (HRQOL)^8^. However, the association of the concentrations of blood heavy metals, including lead, mercury, and cadmium, with developmental delay outcomes has not been studied^9^,^10^. In this study, we proposed 3 hypotheses. First, children with developmental delays have a lower health status and family function than those with typical development. Second, the blood heavy metal concentration is higher in children with developmental delays than in those with typical development. Third, the blood heavy metal concentration is associated with the health status of children.

## Results

Table 1 lists the sociodemographic characteristics of the children and their mothers. In total, we enrolled 92 boys and 86 girls. The developmental delay and typical development groups each consisted of 89 participants with mean ages of 5.87 and 6.15 years, respectively. The developmental delay group consisted of 4 children with cognitive dysfunction, 13 with speech-language delay, 9 with gross and fine motor delay, 5 with social and emotional delay, 40 with global functioning delay, and 18 with other developmental delays, such as sensory integration dysfunction. The participants’ age, sex, birth weight, and body mass index (BMI) did not significantly differ between the 2 groups. The children with developmental delays had a significantly lower number of gestational weeks than those with typical development (37.47 vs. 38.37, P = 0.04). The mothers of the children with developmental delays had significantly higher parity (P = 0.04) and a lower educational level than the mothers of the children with typical development (49.44% vs. 66.29%, P = 0.02). The age, BMI, and gestational age of the mothers did not significantly differ between the 2 groups.

Table 2 lists the results of the parental assessment of the children’s health status and family function. Compared with the children with typical development, those with developmental delays had a significantly poorer HRQOL in physical health (P < 0.01), psychosocial health (P < 0.01), and total health (P < 0.01), and lower performance in upper extremity and physical functioning (P = 0.01), transfer and basic mobility (P = 0.01), sports and physical functioning (P = 0.01), happiness (P < 0.01), and global functioning (P < 0.01). Moreover, compared with the parents of the children with typical development, the parents of the children with developmental delays had a significantly poorer HRQOL in the environmental domain (P = 0.04); a higher family impact score including parent (P < 0.01), family (P < 0.01), and total functioning (P < 0.01); and higher anxiety (P = 0.02).

Table 3 illustrates that blood lead had a significantly positive association with developmental delays of children in a dose-response manner after multivariable adjustment [blood lead 5.00–9.99 μg/dL vs. <5.0 μg/dL, OR = 11.43, 95% confidence interval (CI): 4.69–27.86, p < 0.01; and blood lead ≥10.00 μg/dL vs. <5.0 μg/dL, OR = 14.46, 95% CI: 3.01–69.58, p < 0.01, respectively]. We also analysed using conditional logistic regression, and found the results more efficient (blood lead 5.00–9.99 μg/dL vs. <5.0 μg/dL, OR = 6.17, 95% CI: 2.52–15.11, p < 0.01; and blood lead ≥10.00 μg/dL vs. <5.0 μg/dL, OR = 13.27, 95% CI: 1.62–108.93, p < 0.05, respectively) (data not shown). In addition, we also analysed the association of the blood lead concentration with developmental delays by using the reference cut-off value of 5 μg/dL. The OR of developmental delays was significantly higher for participants with a blood lead concentration of ≥5 μg/dL than for those with a blood lead concentration of <5 μg/dL (OR = 12.04, 95% CI: 5.32–27.26, P < 0.01) (data not shown). The children’s blood mercury concentration was not related to developmental delays. The cadmium concentration was not different between children with developmental delays and those with typical development; however, the OR of developmental delays was significantly higher for participants with a blood cadmium concentration of >1.0 μg/L than for those with a blood cadmium concentration of ≤0.6 μg/L (OR = 2.24, 95% CI: 1.01–5.01, P < 0.05).

In this study, multiple linear regression analysis was used to determine the association of blood lead, mercury, and cadmium concentrations with the children’s health status. Table 4 illustrates the association of heavy metal concentrations with the children’s health status, including HRQOL and functional performance. In all the children, increased blood lead concentrations were significantly associated with decreased HRQOL scores, including physical health (−0.53, P < 0.5), psychosocial health (−0.47, P < 0.5), and total health (−0.48, P < 0.5). Blood mercury and cadmium concentrations were not associated with health status, even in the analysis done based on the children’s developmental status (data not shown).

## Discussion

In this study, we evaluated the association of blood lead, mercury, and cadmium concentrations with developmental delays and health status in Taiwanese children. The blood lead and cadmium concentrations, the number of gestational weeks, parity order, and the mother’s educational level significantly correlated with developmental delays in the children. However, no significant correlation was noted for the blood mercury concentration. The mean blood lead concentration of the children with developmental delays was 7.50 μg/dL, which was significantly higher than that of the children with typical development and higher than the suggested threshold concentration of 5 μg/dL. To the best of our knowledge, this is the first study to demonstrate a dose-response association between the blood lead concentration and developmental delays in children. The children with developmental delays had significantly poorer HRQOL and lower functional performance and family function than did those with typical development. The blood lead concentration was negatively associated with HRQOL in the children. However, health status was unaffected by blood mercury and cadmium concentrations.

In children, the major sources of lead exposure are lead paint, dust, soil, food and beverage, traditional folk remedies, and parental occupational lead exposure^11^. The risk of lead exposure is higher in foetuses and young children than in adults for several reasons^12^. The developing nervous system of children is more sensitive to lead toxicity than the developed brain of adults, and the brain of children aged ≤5 years is particularly susceptible to exposure to lead circulating in the blood^13^. Prenatal and early childhood lead exposure can cause cognitive and language dysfunction that may persist throughout childhood and even into adulthood^14^,^15^,^16^. Studies have reported an association between lead exposure and learning disability; cognitive dysfunction with a decreased intelligence quotient; neuropsychiatric and social behaviour disorders such as attention-deficit hyperactivity disorder, antisocial behaviour, somatic complaints, and aggression; poor neuromotor performance such as poor balance control and eye-hand coordination; and sleep disturbance^17^,^18^. Prenatal and postnatal blood lead concentrations are associated with higher rates of total arrests and arrests for offences involving violence^19^, suggesting a prolonged effect of lead exposure. Therefore, the United States Centers for Disease Control and Prevention has set a blood lead concentration of 5 μg/dL in children as the threshold for action^11^.

In Taiwan, leaded gasoline was believed to be the main source of lead pollution^20^. Since the Environmental Protection Administration banned leaded gasoline in 2000, airborne lead exposure has decreased^21^. In 1994, the mean blood lead concentration in children was 6.0 μg/dL in the less-polluted Penghu Island^22^. After leaded gasoline was banned, the mean blood lead concentration was found to be 5.5 μg/dL in 2002 in children residing in Kaohsiung, an industrial area with high environmental lead exposure^23^. Reports in the recent 5 years have shown that the mean blood lead concentration in children aged 2–12 years ranges from 1.60 to 3.83 μg/dL^15^,^24^. In Taiwan, baseline environmental lead exposure has substantially decreased. However, our data demonstrated a mean blood lead concentration of up to 7.50 μg/dL in children with developmental delays, which was higher than the national threshold concentration of 5 μg/dL. We observed a dose-response association between the blood lead concentration and developmental delays in children. Recent data have shown that even low levels of lead exposure can have irreversible and persistent negative consequences on children’s health^16^,^25^. However, no threshold level has been reported for the adverse effects of lead exposure^16^,^25^. Thus, our results indicate that reducing or preventing children’s exposure to environmental toxins is crucial, particularly for those with developmental delays.

Severe neurological disabilities have been observed in infants born to mothers who consume fish contaminated with high mercury concentrations^26^. Neurodevelopmental and cognitive deficits have been reported in children with prenatal or postnatal low-level mercury exposure^27^,^28^. In addition, a significant association has been reported between the blood mercury concentration and autism spectrum disorders, attention-deficit hyperactivity disorder, and antisocial behaviour^29^,^30^. However, in this study, the blood mercury concentration was not associated with children’s developmental delays and health status. Moreover, our results are consistent with those of previous studies, in which the risk of developmental delays, autism, and behavioural disorders such as emotional and attention disorders^10^,^31^ did not increase after mercury exposure.

Cadmium is an extremely toxic metal commonly found in industrial products, cigarettes, and food contaminants^32^,^33^. Animal studies have revealed that cadmium can cross the blood-brain barrier, and because of its neurotoxicity, cadmium can impede dendritic and synaptic neuron development, inhibit signalling pathways of neurons, alter the neurochemistry of the brain, and induce morphological abnormalities in the cephalic region^32^,^33^,^34^,^35^. In the present study, due to the blood cadmium concentrations of most of children were lower than the national reference value, we selected the tertiles of the controls as the cut-off points. A blood cadmium concentration higher than 1.0 μg/L was found to have a 2-fold greater association with developmental delays in comparison with a blood cadmium concentration of 0.6 μg/L. Some studies have reported that postnatal cadmium exposure is associated with the development of learning disabilities in children and cognitive deficits in boys^4^,^36^. However, a recent meta-analysis reported inconclusive results concerning the effects of cadmium exposure on children’s neuropsychological development^34^. Children are the most affected by the failure to address environmental threats worldwide^37^. Approximately 43% of the disease burden attributable to environmental factors is borne by children aged <5 years^37^. Among these environmental factors, both lead and cadmium exert neurotoxic effects on the developing brain of children^13^,^32^,^33^,^34^,^35^. Although the blood cadmium concentration was not associated with health status in this study, preventing exposure to cadmium and lead is crucial because of their neurotoxicity and the susceptibility of the developing brain of children to these neurotoxic metals.

Information on the status of HRQOL and functional performance can help practitioners, caregivers, the public, and other stakeholders to monitor and treat diseases related to neurotoxic metal exposure and prevent the exposure of children to these neurotoxic metals. In this study, we observed a lower health status and family function in the children with developmental delays than in those with typical development. Moreover, the blood lead concentration was negatively associated with HRQOL in the children. However, blood mercury and cadmium concentrations were not associated with health status, including HRQOL and functional performance. Health is a multidimensional construct that consists of a person’s perception of his or her personal life with regard to various medical conditions and treatments^38^,^39^. The WHO emphasises that health is not merely the absence of an infirmity or a disease but a state of physical, mental, and social well-being^40^. Various factors may affect children’s health status. The blood lead concentration significantly differed between the 2 groups; however, the temporality of exposure in relation to developmental delays remains unclear. Nevertheless, lead, mercury and cadmium are highly neurotoxic to children. Thus, the following measures are crucial to reduce or prevent exposure to these neurotoxic metals: primary prevention of environmental exposure through an evidence-based health policy, vigilant exposure-related history taking, education of families regarding changes in housing, and provision of guidance and resources to families for reducing toxin exposure^14^,^16^,^41^.

This study has several strengths. It is a demographic assessment of the effects of heavy metals on the development of children in a particular geographical and ethnic population. We evaluated the physical function, psychosocial health, and functional performance of the children in the population. We used different high-validity instruments and algorithms to analyse each variable quantitatively.

This study has several limitations. First, the major limitation of our case-control study design is that exposure (blood levels) was measured after the outcome (developmental delays), which may have been present since birth or even before birth. Therefore, causation cannot be inferred without information on exposure at an early age. Moreover, an association may exist between heavy metal exposure and developmental delays; however, exposure may be a consequence (and not a cause) of developmental delay (e.g., pica-type behaviour and increased hand-to-mouth behaviour). Furthermore, developmental delay itself may be a more appropriate causal factor affecting children’s health status than heavy metal concentrations. Therefore, the constraint of the exposure-outcome timeline in the present study may have confounded the results related to the association of heavy metal concentrations with the children’s health status. This is a crucial point and may affect the validity and interpretation of our data. Second, controls were considered to have typical development on the basis of regular physical check-ups and developmental screening conducted by paediatric doctors at clinics. Because of limited financial support and manpower restrictions, we could not administer to the controls the tests for detecting the presence of developmental delays that were administered to the children with suspected developmental delays. Therefore, we could not provide data demonstrating that the controls were true controls by using descriptive statistics and ranges of test scores for the case versus the control groups. Third, the developmental disorders of the children varied because they were recruited from clinics for children with developmental delays. Although our results cannot be extended to children in a more specific group, such as those with only a speech-language delay, the results accurately represent children assessed and treated in clinical settings. Fourth, we used translated questionnaires that have shown reliability in cross-cultural assessments of HRQOL^7^,^42^. However, the existence of cross-cultural differences cannot be excluded. Fifth, proxy reports are often used for HRQOL assessment in young children because they may not have adequate cognitive ability and communication skills to understand and respond to abstract constructs^38^. Nevertheless, some differences may exist between parents’ and children’s viewpoints regarding HRQOL, which may have affected the results of evaluations based on parent-reported questionnaires. Finally, the samples were collected from Taiwanese children. Therefore, whether our results can be extended to children of other ethnicities remains uncertain. Studies sampling children of different ethnicities and with a larger sample size should be conducted to generalise the results.

## Conclusions

We demonstrated that the blood lead and cadmium concentrations, the number of gestational weeks, parity order, and the mother’s educational level were significantly associated with developmental delays in children. However, we noted no significant association for mercury concentration. The children with developmental delays had a higher blood lead concentration than did those with typical development, and the blood lead concentration was higher than the national suggested threshold of 5 μg/dL. A dose-response association was observed between the blood lead concentration and developmental delays in the children. Moreover, this concentration was negatively associated with the children’s HRQOL. The children with developmental delays had a significantly poorer HRQOL and lower functional performance and family function than those with typical development. Because of the neurotoxicity of heavy metals, the primary prevention of environmental toxin exposure is crucial in children.

## Methods

### Participants

This case-control study was conducted between March 2010 and December 2014 at Shin Kong Wu Ho-Su Memorial Hospital, a medical centre with 872 beds in Northern Taiwan. Children with suspected developmental delays from Taipei City and New Taipei City were referred by paediatric clinics, kindergartens, community centres, and primary elementary schools. The developmental intervention team at the hospital consisted of a physiatrist, a paediatrician, occupational therapists, speech therapists, physical therapists, a psychologist, a psychiatrist, an otolaryngologist, an ophthalmologist, and a social worker. All patients underwent a developmental assessment to confirm the presence of developmental delays, including those in speech-language, social, cognition, gross motor, fine motor, and emotional domains. Developmental delay was defined as a performance score of ≥2 standard deviations lower than the mean in age-appropriate, standardised, norm-referenced developmental tests. Our previous study presented the detailed procedure^42^.

In total, we recruited 89 children diagnosed with developmental delays and their parents from the hospital’s developmental intervention clinic. In addition, we recruited 89 age (**±**5 years)- and sex-matched children with typical development who underwent regular developmental screening at the Department of Paediatrics during the same period, and we used their parents as controls. This study was approved by the Research Ethics Committee of Shin Kong Wu Ho-Su Memorial Hospital, and was performed in accordance with the World Medical Association Declaration of Helsinki. All parents provided written informed consent for them and their children to participate in this study. This study was registered at ClinicalTrials.gov (NCT02523989, date of registration: Aug. 13, 2015).

### Questionnaire interview

A well-trained examiner administered structured questionnaires to the parents during interviews at the clinic. In addition, information was obtained on the demographics of the children and their parents and on the health status of the children.

### Health status of children

The Pediatric Quality of Life (PedsQL) Inventory Generic Core Scales (parent proxy-report format) were used to measure paediatric HRQOL according to the parents^43^. Multiple dimensions, namely physical, social, emotional, and school functioning, were evaluated, and physical, psychosocial, and total scale scores were calculated. The scores ranged from 0 to 100, with 100 being the highest level of HRQOL. The Chinese version of the PedsQL Generic Core Scales demonstrated satisfactory reliability (interrater reliability: 0.45–0.80; intrarater reliability: 0.62–0.81), validity, and feasibility^44^. A Pediatric Outcomes Data Collection Instrument (PODCI) parent questionnaire was used to assess the children’s functional performance^45^. The PODCI evaluated patients based on the following 6 scales: upper extremity and physical functioning, transfer and basic mobility, sports and physical functioning, pain and comfort, happiness, and global functioning. The score ranged from 0 to 100, with a higher score indicating a more satisfactory functional performance. The Chinese version of the PODCI exhibited satisfactory reliability (interrater reliability: 0.97; intrarater reliability: 0.83)^46^,^47^.

### Family function

Family function was evaluated using 3 instruments. First, the Chinese version of the WHO Quality of Life-Brief Version questionnaire^48^, which comprises the physical health, psychological and social relationship, and environmental domains, was used to evaluate family function. The scores ranged from 0 to 100, with a higher score indicating a higher HRQOL. This questionnaire exhibited satisfactory reliability (interrater reliability: 0.97; intrarater reliability: 0.88)^47^,^48^. Second, the Chinese version of the PedsQL Family Impact Module^43^, which exhibited satisfactory reliability (interrater reliability: 0.79; intrarater reliability: 0.97)^47^, was used to assess the family impact. The PedsQL Family Impact Module yielded the parental functioning summary score (physical, emotional, cognitive, and social functioning; communication; and worry domains), the family functioning summary score (daily activity and family relationship domains), and the total score. The scores ranged from 0 to 100, with higher scores indicating a lower family impact. Third, the Hospital Anxiety and Depression Scale (HADS) was used to evaluate parental psychological distress. An HADS score of >7 indicated the presence of anxiety or depression^49^. The Chinese version of the HADS score exhibited fair reliability (interrater reliability: 0.79; intrarater reliability: 0.99)^47^.

### Biological specimen collection and blood lead, mercury, and cadmium concentration measurements

We sampled 5–8 mL of blood from the median cubital or cephalic veins. The blood was collected in a vacuum tube containing ethylenediaminetetraacetic acid. The separated red blood cells were frozen at −80 °C within 3 months to analyse the heavy metals concentration. The red blood cells were thawed at room temperature and then digested using nitric acid in a microwave (Perkin Multiwave 3000). Blood lead, mercury, and cadmium concentrations were measured using inductively coupled mass spectrometry (Thermo X-series II). Seronorm^TM^ Trace Elements Whole Blood, which has certified lead, mercury, and cadmium concentrations of 31.0 μg/dL (range: 28.6–33.4 μg/dL), 16.0 μg/L (range: 9.6–22.4 μg/L), and 5.8 μg/L (range: 5.4–6.2 μg/L), respectively, was used as a control for assessing the validity of the measurements. The lead, mercury, and cadmium concentrations of Seronorm^TM^ Trace Elements Whole Blood were measured as 32.9 ± 1.7 μg/dL, 20.4 ± 2.4 μg/L, and 6.1 ± 0.5 μg/L, respectively, in our system. The detection limits of lead, mercury, and cadmium were 0.03 μg/dL, 0.50 μg/L, and 0.07 μg/L, respectively.

### Statistical analysis

The results were expressed as the mean ± standard error. The chi-squared test or the *t* test was used to analyse the demographic data of both groups. Multivariate logistic regression models were used for estimating the ORs and 95% CI to determine the association of blood heavy metal concentrations with developmental delays after adjustment for age, sex, and the number of gestational weeks, parity order, and the mother’s educational level. The cut-off points for blood cadmium levels were the respective tertiles of the controls, and the cut-off points for blood lead were 5.00 and 10.00 μg/L in the dose-response analysis. Blood mercury concentration was categorized by the reference level of 5.80 μg/L^50^. Significance tests for the linear trend among ORs across exposure strata were calculated by categorising exposure variables and treating scored variables as continuous. Multiple linear regression analysis was used to calculate the regression coefficient and 95% CIs to determine the association of blood heavy metal concentrations with health status (PedsQL score and PODCI score) after adjustment for age, sex, the number of gestational weeks, parity order, the mother’s education level, and other blood metals. All analyses were conducted using the Statistical Analysis Software package, Version 8.0 (Cary, NC, USA). The level of statistical significance was set at P < 0.05 (2-sided). The sample size was calculated by assuming a standardised effect (mean difference/standard deviation) of 0.5. To achieve 90% power at a significance level of 0.05, each group had to include at least 86 participants to evaluate the association of blood heavy metal concentrations with developmental delays^6^.

## Additional Information

**How to cite this article**: Hsueh, Y.-M. *et al*. Association of blood heavy metals with developmental delays and health status in children. *Sci. Rep.* **7**, 43608; doi: 10.1038/srep43608 (2017).

**Publisher's note:** Springer Nature remains neutral with regard to jurisdictional claims in published maps and institutional affiliations.

## Figures and Tables

**Table 1:** Sociodemographic characteristics of children and their mothers.

Variables	Children with developmental delays (n = 89)	Children with typical development (n = 89)	P value
Children			
Age (years)	5.87 ± 0.19	6.15 ± 0.29	0.43
BMI (kg/m^2^)	15.93 ± 0.35	16.43 ± 0.36	0.32
Birth weight (g)	2976.95 ± 71.74	3101.90 ± 58.64	0.18
Sex			
Male	46 (51.69)	46 (51.69)	1.00
Female	43 (48.31)	43 (48.31)	
Mothers			
Age (years)	35.68 ± 0.55	36.34 ± 0.56	0.36
BMI (kg/m^2^)	22.81 ± 0.49	22.65 ± 0.40	0.79
Gestational age (years)	29.76 ± 0.58	30.11 ± 0.54	0.65
Gestational weeks	37.47 ± 0.37	38.37 ± 0.23	0.04
Parity			
One	42 (47.19)	52 (58.34)	0.04
Two	35 (39.33)	34 (38.20)	
Three or more	12 (13.48)	3 (3.37)	
Educational level			
High school or lower	45 (50.56)	30 (33.71)	0.03
College or higher	44 (49.44)	59 (66.29)	

BMI: body mass index. Values are expressed as the number (percent) or mean ± SE unless noted otherwise.

**Table 2:** Health status and family function of children.

Variables	Children with developmental delays		Children with typical development		P value
	N	Mean ± SE	N	Mean ± SE	
Health status of children					
HRQOL: PedsQL					
Physical health	89	72.23 ± 2.56	89	93.15 ± 1.16	<0.01
Psychosocial health	89	66.74 ± 1.83	89	85.11 ± 1.60	<0.01
Total health	89	68.05 ± 1.87	89	87.01 ± 1.41	<0.01
Function: PODCI					
Upper extremity and physical functioning	81	85.96 ± 1.80	86	93.94 ± 1.10	<0.01
Transfer and basic mobility	81	93.98 ± 1.69	86	98.56 ± 0.49	0.01
Sports and physical functioning	81	88.92 ± 1.84	86	94.93 ± 1.10	0.01
Pain and comfort	81	85.00 ± 1.64	86	87.10 ± 1.59	0.36
Happiness	81	78.83 ± 1.65	86	89.83 ± 1.35	<0.01
Global functioning	81	86.68 ± 1.34	86	92.60 ± 0.78	<0.01
Family function					
HRQOL: WHOQOL-BREF					
Physical health	87	45.72 ± 1.04	85	46.76 ± 1.22	0.52
Psychological	87	50.13 ± 1.39	85	51.19 ± 1.32	0.58
Social relationships	87	58.45 ± 1.65	85	62.14 ± 1.56	0.11
Environment	87	53.59 ± 1.54	85	57.94 ± 1.45	0.04
Family impact: PedsQL Family Impact Module					
Parent	89	66.78 ± 2.16	89	78.76 ± 2.02	<0.01
Family	89	61.31 ± 2.29	89	74.81 ± 2.38	<0.01
Total	89	65.12 ± 2.02	89	86.88 ± 1.97	<0.01
Psychological distress: HADS					
Anxiety	89	6.65 ± 0.40	88	5.61 ± 0.44	0.08
Normal		53 (59.55)		67 (76.14)	0.02
Abnormal		36 (40.45)		21 (23.86)	
Depression	89	6.45 ± 0.41	88	5.13 ± 0.38	0.02
Normal		56 (62.92)		62 (70.45)	0.29
Abnormal		33 (37.08)		26 (29.55)	

Values are expressed as the number (percent) or mean ± SE unless noted otherwise.

HRQOL: health-related quality of life; PedsQL: Pediatric Quality of Life Inventory Generic Core Scales; PODCI: Pediatric Outcomes Data Collection Instrument; WHOQOL-BREF: World Health Organization

Quality of Life-Brief Version; PedsQL Family Impact Module: Pediatric Quality of Life Inventory Family Impact Module; HADS: Hospital Anxiety and Depression Scale.

**Table 3:** Dose–response association of blood metal concentrations with the risk of developmental delays in children.

Variables	Children with developmental delays (n = 89)	Children with typical development (n = 89)	Multivariate ORs (95% CI)^a^
Lead (μg/dL)	7.50 ± 0.85	3.57 ± 0.22	1.54 (1.30–1.83)^**^
<5.00	34 (38.20)	79 (88.76)	1.00^§^
5.00–10.00	39 (43.82)	8 (8.99)	11.43 (4.69–27.86)^**^
≥10.00	16 (17.98)	2 (2.25)	14.46 (3.01–69.58)^**^
Mercury (μg/L)	6.83 ± 0.68	9.58 ± 2.36	0.98 (0.94–1.03)
<5.80	50 (56.18)	39 (43.82)	1.00
≥5.80	39 (43.82)	50 (56.18)	0.55 (0.29–1.03)
Cadmium (μg/L)	1.19 ± 0.09	1.34 ± 0.16	0.89 (0.69–1.13)
≤0.60	20 (22.47)	30 (33.71)	1.00
0.60–1.00	29 (32.58)	31 (34.83)	1.82 (0.81–4.08)
>1.00	40 (44.94)	28 (31.46)	2.24 (1.01–5.01)^*^

Values are expressed as the number (percent) or mean ± SE unless noted otherwise.

^a^Adjusted for age, sex, the number of gestational weeks, parity order, and the mother’s educational level. +0.05 ≤ P < 0.1. ^*^P < 0.05. ^**^P <^ ^0.01. ^§^P < 0.05 for the trend test.

**Table 4:** Association of blood heavy metal concentrations with the health status of children.

Variables	All children (n = 178)		
	Lead (μg/dL)	Mercury (μg/L)	Cadmium (μg/L)
HRQOL: PedsQL			
Physical health	−0.53 (−1.04 to −0.02)^*^	0.06 (−0.13 to 0.25)	1.02 (−1.48 to 3.52)
Psychosocial health	−0.47 (−0.92 to −0.01)^*^	0.13 (−0.04 to 0.30)	0.99 (−1.22 to 3.19)
Total health	−0.48 (−0.92 to −0.04)^*^	0.11 (−0.05 to 0.28)	1.02 (−1.13 to 3.18)
Function: PODCI			
Upper extremity and physical functioning	0.16 (−0.27 to 0.59)	0.06 (−0.06 to 0.19)	0.88 (−0.78 to 2.55)
Transfer and basic mobility	−0.03 (−0.39 to 0.32)	0.03 (−0.07 to 0.13)	0.39 (−0.97 to 1.76)
Sports and physical functioning	−0.22 (−0.64 to 0.21)	0.05 (−0.08 to 0.17)	0.43 (−1.21 to 2.06)
Pain and comfort	−0.09 (−0.56 to 0.37)	−0.06 (−0.19 to 0.07)	−1.59 (−3.38 to 0.21)^+^
Happiness	−0.42 (−0.87 to 0.04)^+^	0.08 (−0.05 to 0.22)	0.22 (−1.56 to 2.01)
Global functioning	−0.15 (−1.64 to 1.33)	0.03 (−0.09 to 0.15)	−0.23 (−2.15 to 1.70)

HRQOL: health-related quality of life; PedsQL: Pediatric Quality of Life Inventory Generic Core Scales; PODCI: Pediatric.

Outcomes Data Collection Instrument.

Values are expressed as β (95% CI). β, regression coefficient; CI, confidence interval.

Adjusted for age, sex, the number of gestational weeks, parity order, the mother’s educational level, and other blood metals. +0.05 ≤ P < 0.1. ^*^P < 0.05.
